# Supplementary figures and images for: A cysteine-rich domain of the Cryptococcus neoformans Cuf1 transcription factor is required for high copper stress sensing and fungal virulence
Source: mBio. 2026 Jun 9;17(7):e00781-26. doi: 10.1128/mbio.00781-26 (PMC13343922; doi:10.1128/mbio.00781-26)

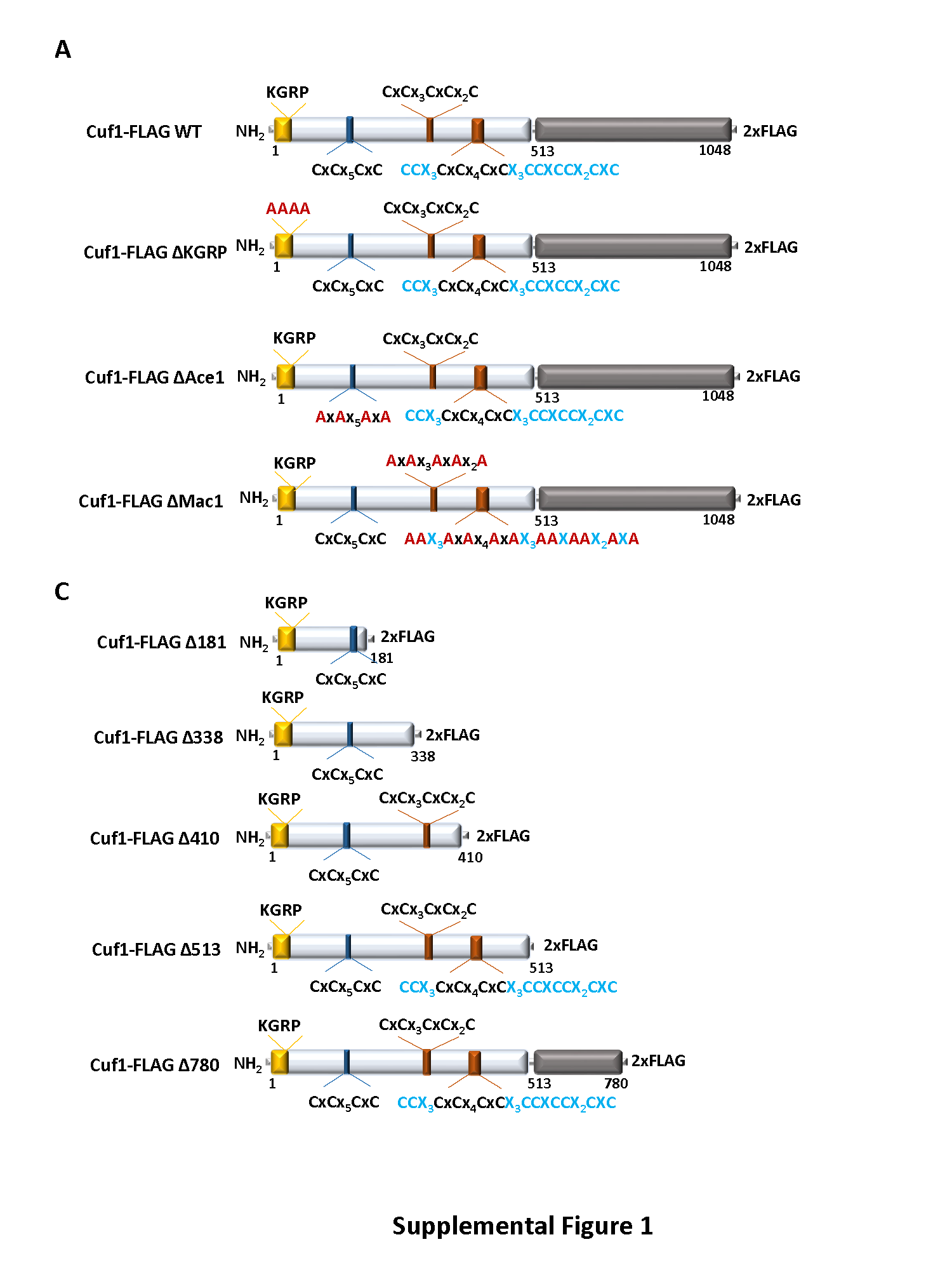

Supplement: Fig. S1 — Schematic representation of all generated Cuf1-FLAG motif mutants and truncations. [file mbio.00781-26-s0001.tif]

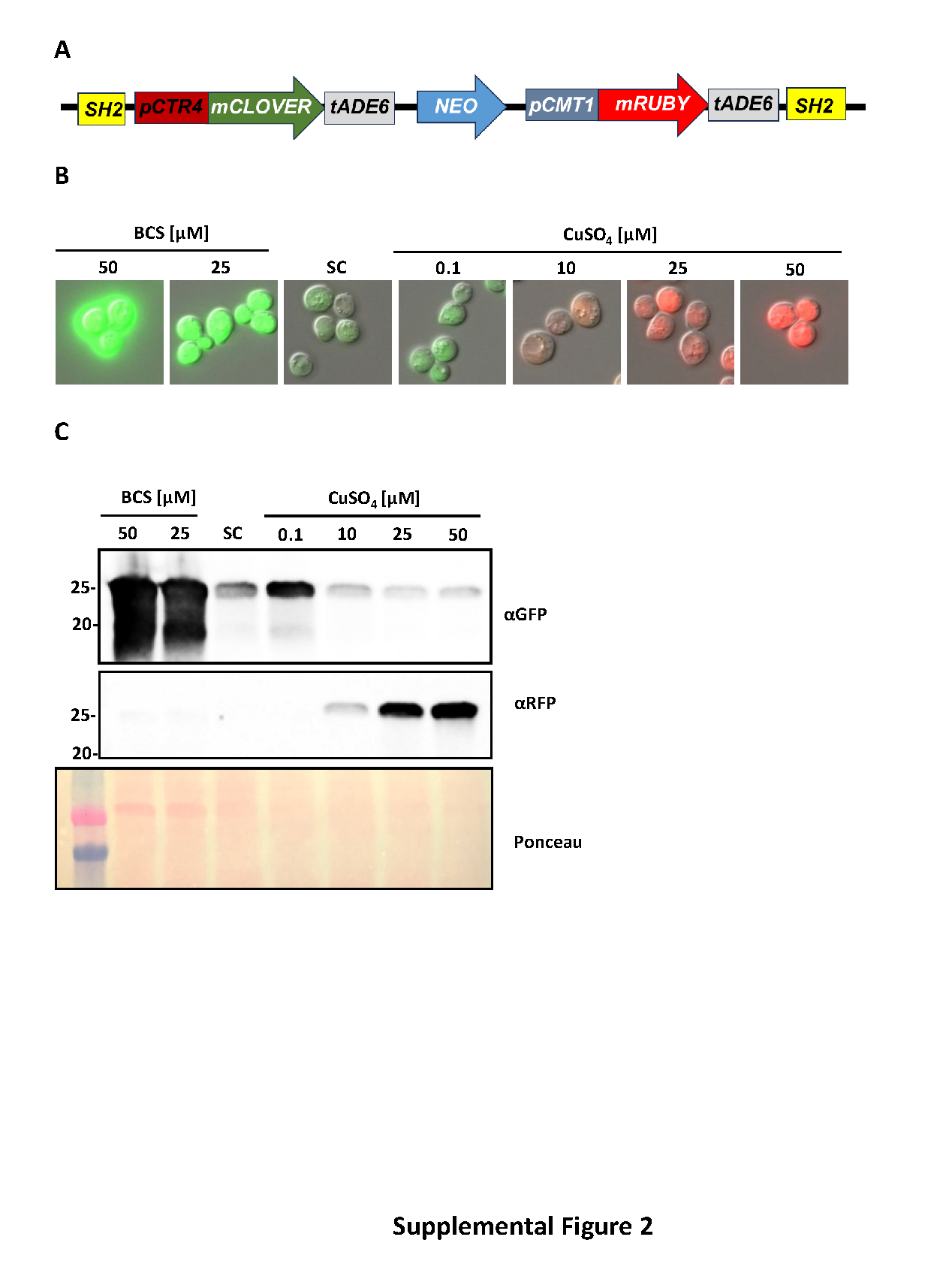

Supplement: Fig. S2 — Schematic representation of strain design and validation of the Cu sensor strain. [file mbio.00781-26-s0002.tif]

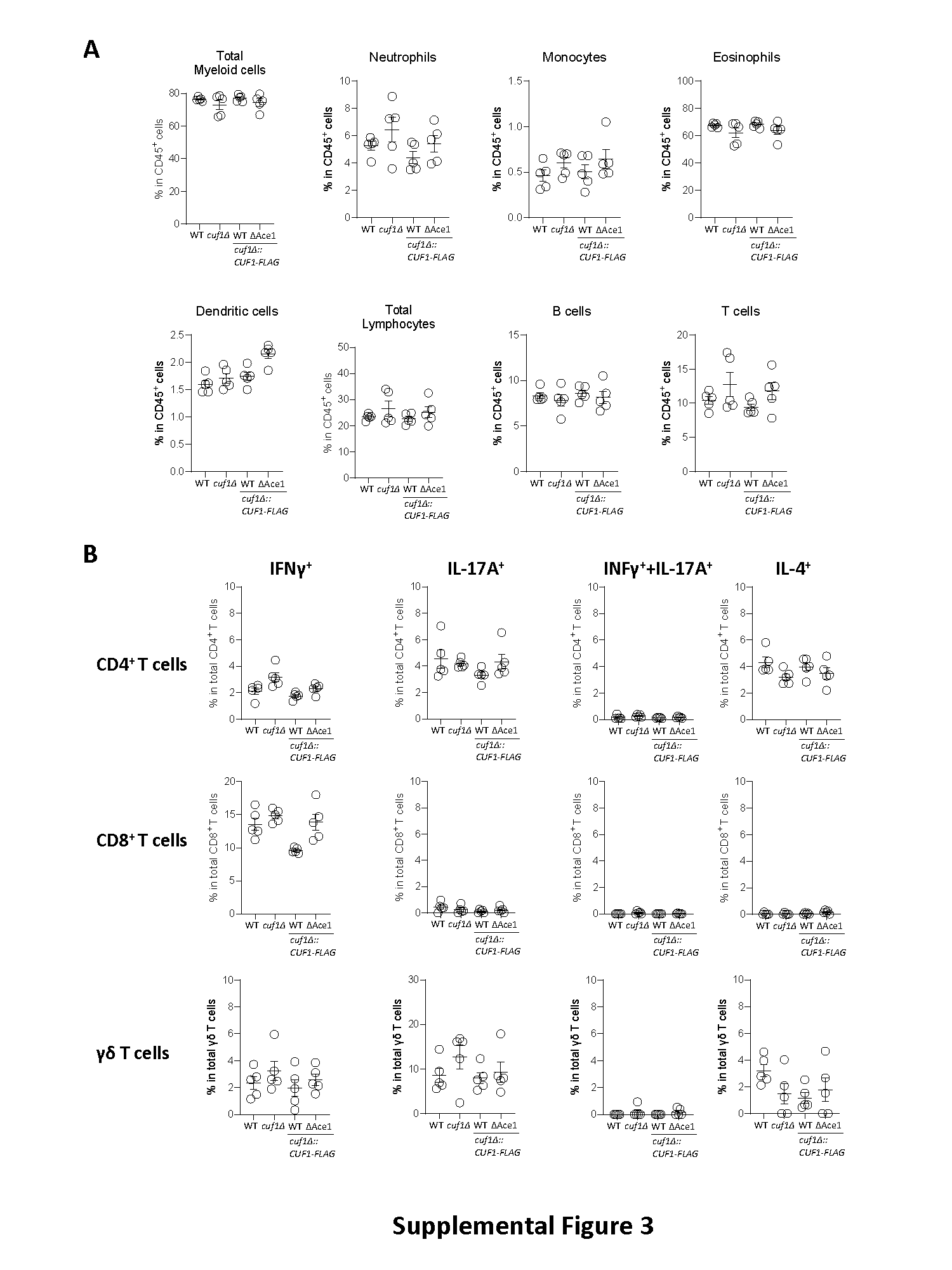

Supplement: Fig. S3 — Flow cytometry analysis of changes in various immune cell populations and T-cell activation. [file mbio.00781-26-s0003.tif]

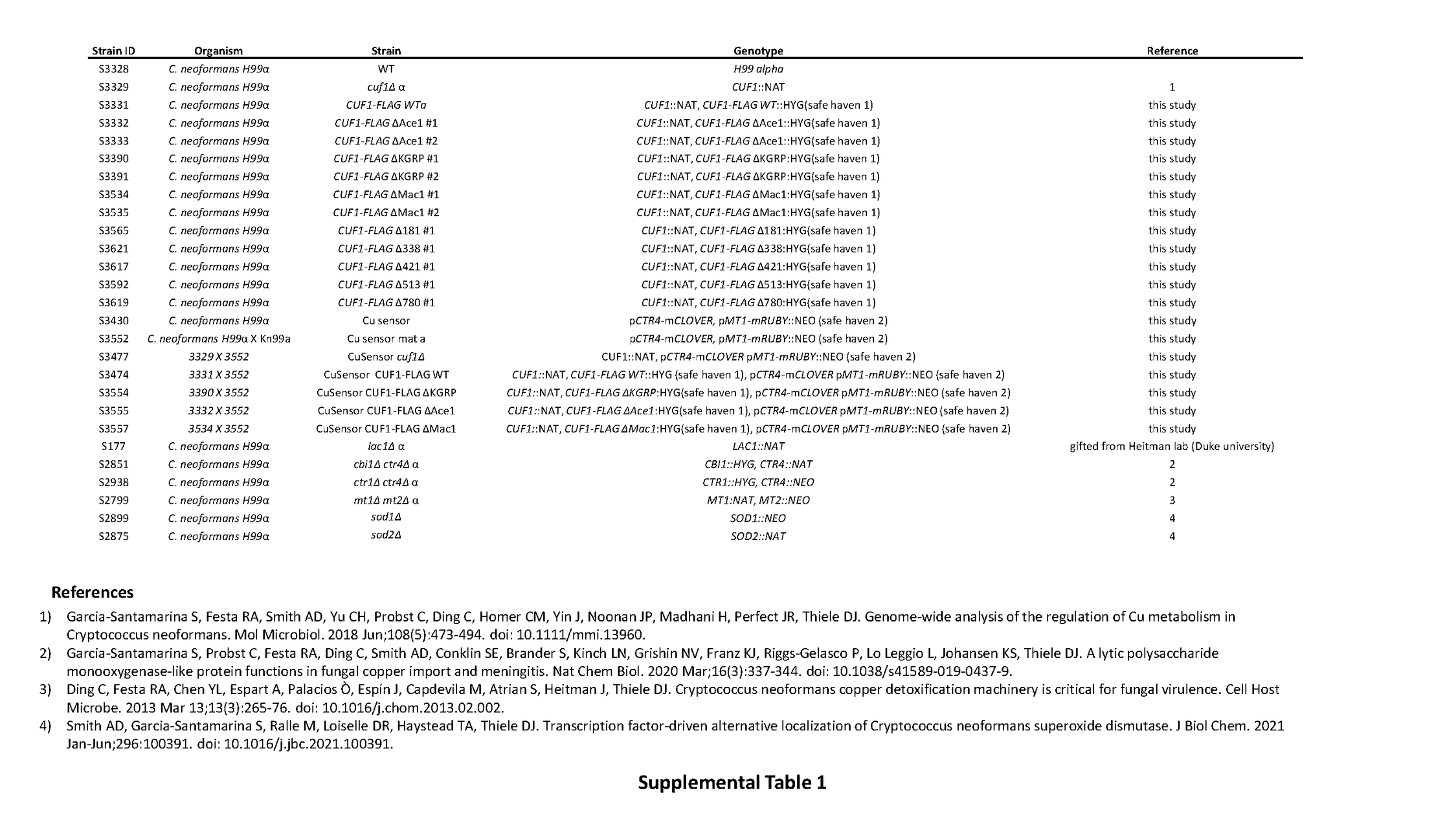

Supplement: Table S1 — Strains used in this study. [file mbio.00781-26-s0005.tif]

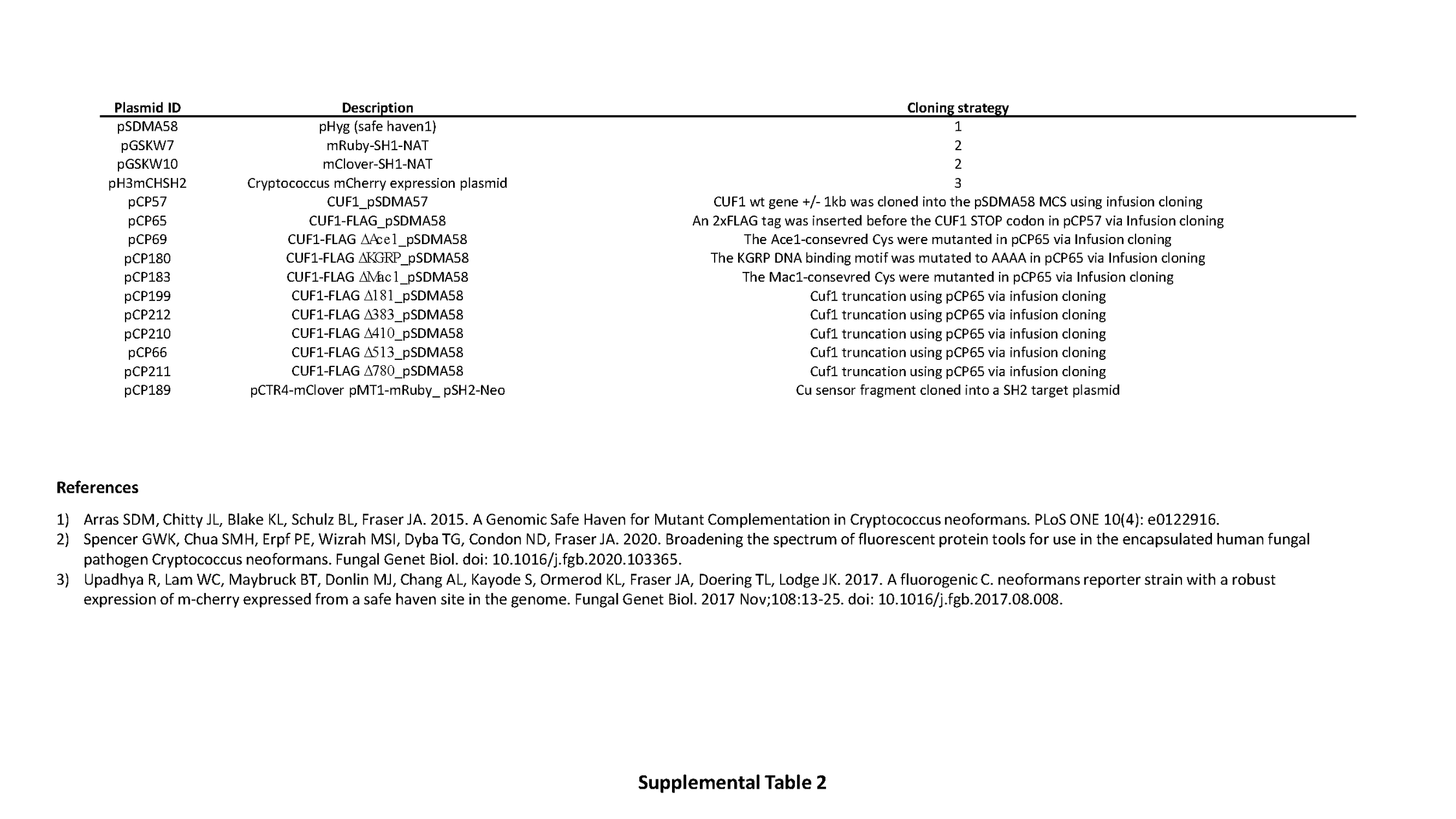

Supplement: Table S2 — Plasmids used in this study. [file mbio.00781-26-s0006.tif]

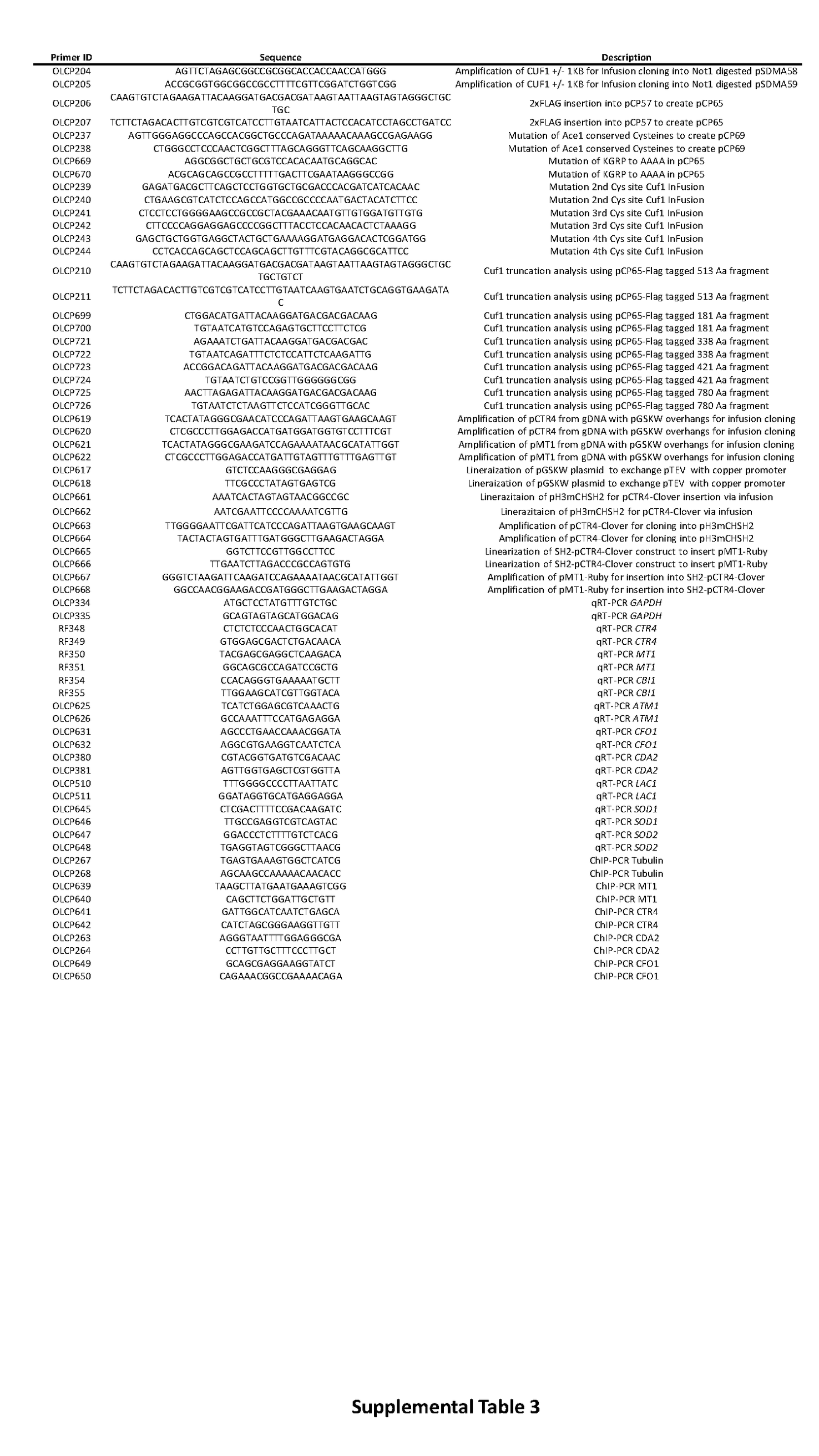

Supplement: Table S3 — Oligonucleotides used in this study. [file mbio.00781-26-s0007.tif]

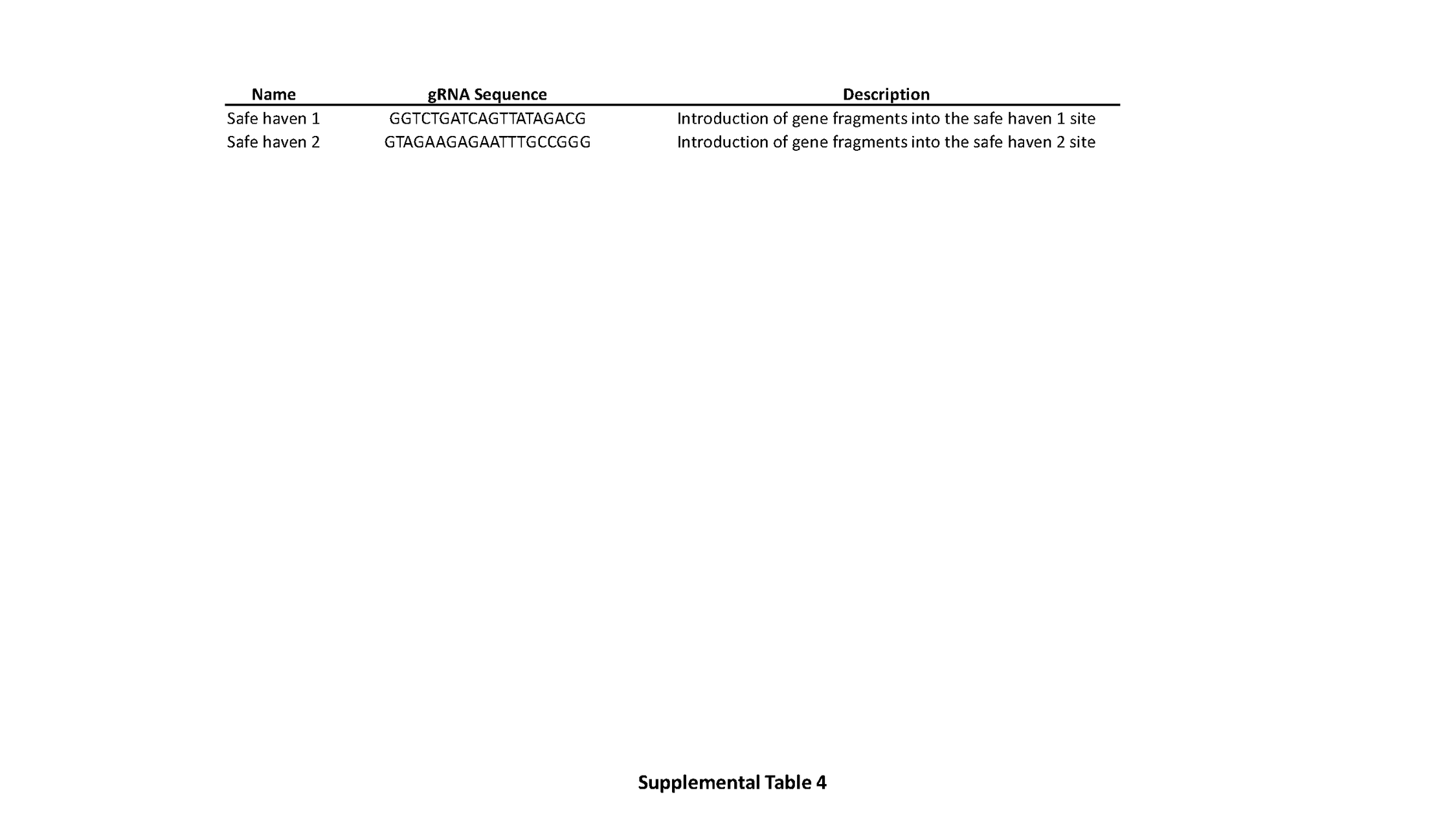

Supplement: Table S4 — Guide RNA used for CRISPR/Cas9-mediated transformation. [file mbio.00781-26-s0008.tif]

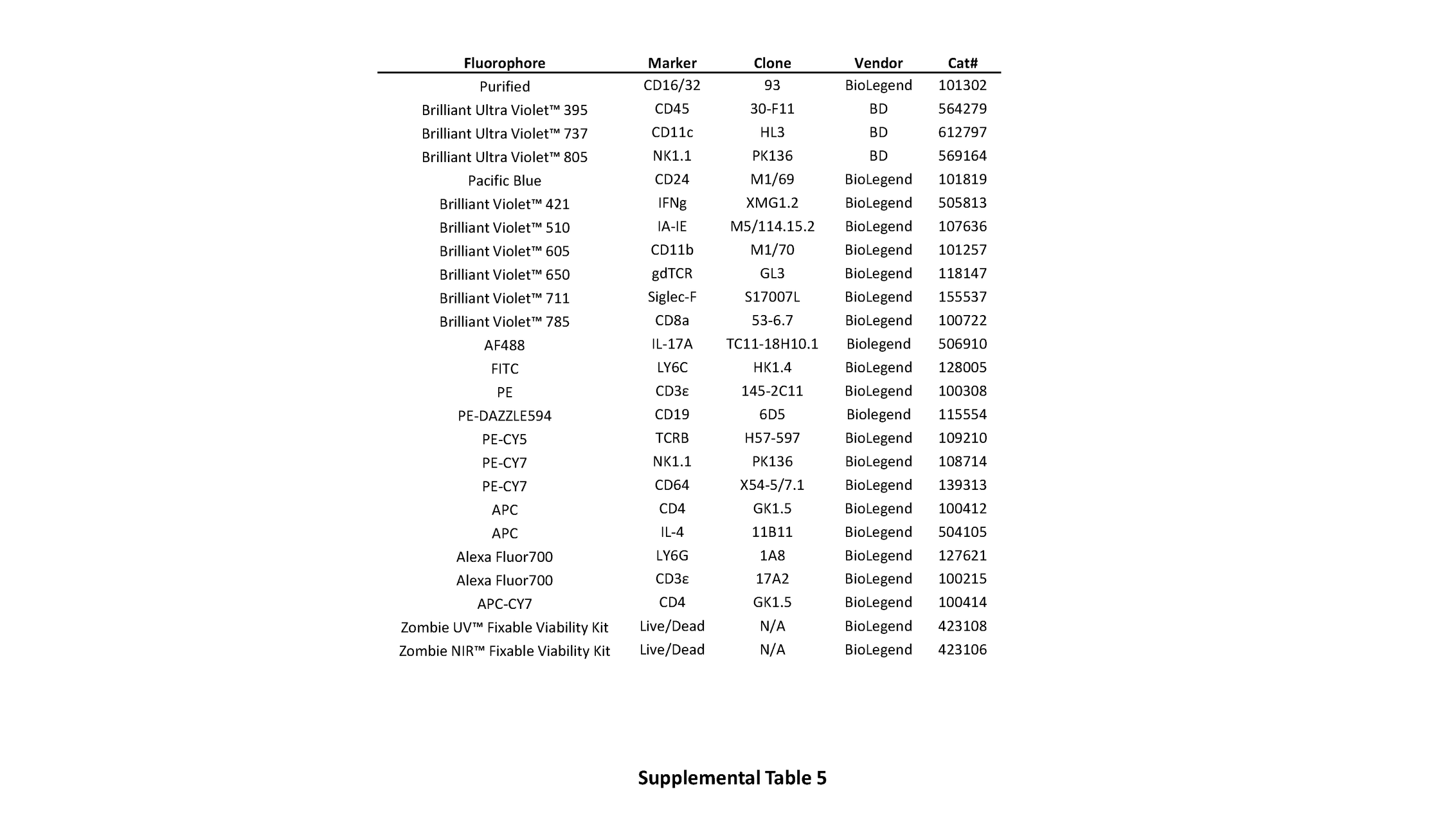

Supplement: Table S5 — Antibodies used for flow cytometry analysis. [file mbio.00781-26-s0009.tif]
